# Judgments of Direction About Words and Arrows: The Neglected Influence of Task Set

**DOI:** 10.1177/17470218261423867

**Published:** 2026-02-03

**Authors:** Derek Besner, Robert S. McCann, Torin P. Young, Kate Van Kessel, Colin M. MacLeod

**Affiliations:** 1 Department of Psychology, University of Waterloo, ON, Canada

**Keywords:** direction judgment, congruency, S–R compatibility, task set

## Abstract

Two experiments investigated the role of task set in processing the direction indicated by arrows and words. In Experiment 1, in one block of trials, participants made stimulus–response (S–R) compatible manual responses to a left-or-right-pointing arrow in the presence of either a congruent or incongruent word (LEFT/RIGHT); in another block, they responded to the direction signaled by a word in the presence of a congruent or incongruent arrow. Task set was, therefore, simple in each block—respond to only one of the two stimulus types. Yet an incongruent word did not interfere with responding to an arrow’s direction, whereas an incongruent arrow robustly interfered with responding to the direction signaled by a word. In Experiment 2, targets were instead defined in terms of a fixed location (for one group, the distractor appeared below the target; for another group, the distractor appeared above the target) such that the target on any trial could be either a word or an arrow. Task set now was more complex in that target type varied unpredictably across trials. Distractor words now interfered with arrows, and distractor arrows interfered with words more than they did in Experiment 1. These results require a more integrative account that embeds S–R compatibility within a larger framework involving contextually driven modulation of task set.

The experiments reported here are extensions of a previous line of research by Besner et al. (2021) that examined interference effects in processing familiar stimuli that designate direction. By way of background, it has long been maintained that the determination of eye-gaze direction is reflexive and informationally encapsulated (see Besner et al., 2021 for a brief summary of such claims that go back at least to Simmel, 1921). Some even argue that there is an innate component (Batki et al., 2000) although it is certainly plausible that repeated exposure in early life results in extensive learning, given the social value of eye gaze. In contrast, the determination of arrow direction is neither considered to be reflexive, nor is it informationally encapsulated, and it is certainly not innate. The question concerned the outcome when these two types of directional stimuli co-occurred with the requirement to respond to one stimulus type and to ignore the other.

Besner et al. (2021) reported a series of 10 experiments in which schematic eyes and arrows were displayed together and were either congruent or incongruent with each other in terms of the direction that they signaled. Based on the literature that they reviewed, it is widely assumed that eyes ought to be processed more automatically than arrows (for more on the concept of automaticity, see Moors & De Houwer, 2006). As a result, their prediction was that distractor eyes would interfere strongly with arrows whereas distractor arrows would interfere weakly, if at all, with schematic eyes. This proved decidedly not to be the case; in fact, the reverse was true. When an explicit judgment was required as to whether arrows pointed left or right, incongruent eyes interfered only very weakly; when the requirement was instead to respond to the direction of eye gaze, incongruent arrows interfered strongly.^1^

The experiments reported here extend the Besner et al. (2021) work with eyes and arrows to a related case—that of words and arrows. Experience with processing common words vastly outweighs experience with processing arrows (indeed, visual word identification is widely viewed as the quintessential example of a strongly automatic process; in that processing of words interferes with other concurrent processes but is itself immune from interference by other processes (e.g., see Neely, 1977; Tse & Neely, 2007 in the case of semantic priming in visual word identification, and the voluminous Stroop literature where it is widely accepted that irrelevant words interfere with color identification whereas irrelevant colors typically do not interfere with word identification; see MacLeod, 1991, for a review). This view leads to the straightforward prediction that when the word LEFT or the word RIGHT is presented together with an arrow pointing left or right, the task of responding to the arrow will display strong interference from an incongruent distractor word. The converse, the task of responding to the word in the presence of an arrow, should show considerably less interference, if any. What does the literature have to say about this seemingly straightforward prediction?

In fact, the results of Baldo et al. (1998) sharply contradict this expectation. In their experiments, when responding was done manually (as in Besner et al., 2021), incongruent arrows interfered strongly with directional decisions about words whereas incongruent words did not measurably interfere with judging the directions of arrows. Baldo et al. did not explicitly consider the implications of their results for the automaticity of visual word recognition. Instead, following Virzi and Egeth (1985), they offered an elegant stimulus–response (S–R) compatibility account of the observed asymmetry in interference. Their account rested on two main assumptions: (a) responding to an arrow with a key press on the side of the keyboard indicated by the direction of the arrow is highly S–R compatible, and (b) responding to a word with a key press on the side of the keyboard indicated by the meaning of the word is more arbitrary, and therefore not S–R compatible—or at least considerably less so.^2^

More specifically, Baldo et al. (1998) claimed that processing the arrow activates the corresponding manual response code directly, rapidly, and strongly. This is consistent with the idea of automaticity without extensive practice (Logan & Etherton, 1994; Yamaguchi & Proctor, 2011): As long as there is an instance of a relevant past event that can be retrieved, responding can occur rapidly and without detailed online analysis. As a result of this high arrow-to-manual response compatibility, when the response must instead be made to the word as target, the incongruent arrow causes substantial interference. In contrast, processing the word activates the manual response code associated with the meaning of the word only indirectly, slowly, and weakly. Consequently, processing the word is dependent on a task set in working memory that contains the necessary S–R translation rules, a task set formed on the basis of the instructions (Liefooghe et al., 2012). Due to the low S–R compatibility between the word and the manual response, an incongruent word should cause virtually no interference when responding to the arrow target. They attributed the small amount of unpredicted interference that they observed when arrows were the targets and words were the distractors (14 ms, *p* = .06) to the sensitivity of their experiments and particularly to the inclusion of target-only trials that likely incremented overall interference across trials.

Additional evidence for their S–R compatibility account emerged from another block of trials where participants responded either to arrow direction or to word meaning by saying the word LEFT or RIGHT aloud instead of by pressing left or right keys. These oral responses to words were largely unaffected by arrow identity, whereas arrows now suffered strong interference from word meaning. This reversal of the interference pattern was seen as indicating that phonological responses were highly S–R compatible with words, whereas the mapping of arrow direction onto a phonological code was now more arbitrary, requiring access to the S–R rules held in working memory. In short, Baldo et al. (1998) viewed S–R compatibility as playing the dominant role in generating the asymmetric interference patterns in these directional interference tasks.

## The Present Study

We report two experiments investigating how the participant comes to respond to the relevant aspect of the stimulus dimension and avoids responding to the irrelevant dimension. The notion of a “task set” figures prominently in answers to this question (Allport et al., 1994; Besner & Care, 2003; Haazebroek et al., 2017; Logan & Bundesen, 2004; O’Malley & Besner, 2014; Rogers & Monsell, 1995). Essentially, a task set is a mental file containing a task-specific store of knowledge, such as the attributes (dimensions) of a stimulus that are relevant for task processing and the S–R translation rules that map values of the relevant attributes onto relevant responses. Performance in multi-tasking environments featuring bivalent stimuli is thus highly dependent on instantiation of the correct task set. When Trial *N* and Trial *N* *+* *1* invoke the same task set, as under blocked conditions, responding is straightforward due to the simple, consistent task set. But under random conditions, when Trial *N* *+* *1* involves a switch in task relative to Trial *N*, the task set instantiated for Trial *N* has to be replaced by the task set appropriate for Trial *N* *+* *1*. What would be the consequence of this more complex task set situation for the interference observed? It is surprising that little attention has been paid to the situation when word identification forms part of the task set.

Experiment 1 set out simply to replicate the manual responding findings of Baldo et al. (1998). There were three notable differences in procedure: (a) we did not include target-only trials given their concern about such trials incrementing overall interference, (b) we did not vary predictability of target location, which they found not to interact with the main variables of interest, and (c) we did not include blocks of vocal responding, given our primary interest in the manual case. Otherwise, our Experiment 1 was very similar to their Experiment 1. On each trial, the stimulus consisted of an arrow pointing to the right or left next to the word LEFT or RIGHT, with the word and arrow being either congruent or incongruent. In one block of trials, a lateralized S–R compatible key press response was to be made to the direction indicated by the arrow. In the other block of trials, a key press response was to be made to the word which is less S–R compatible. When trials are blocked in this manner, the task set—the rule for responding—is simple and straightforward in each block. We expected to replicate the results of Baldo et al., finding that irrelevant arrows interfere with responding to words, but that irrelevant words do not interfere reliably with responding to arrows, despite this pattern being counter to expectations based on the differential experience with the two types of stimuli.^3^

We intended that this replicated pattern would then serve as the departure point for our Experiment 2 in which the context was changed. In our second experiment, task—whether to respond to words or arrows on each trial—was no longer blocked but instead depended on which stimulus appeared at the top (or bottom, depending on the participant group) of the display, which could be either a word or an arrow. We expected this added uncertainty regarding task to slow responding overall and to increment interference for both types of stimuli. A more complicated task set now had to be held in working memory, rather than simply consistently responding to one of the two types of stimuli (either words or arrows) as was the case in Experiment 1. We therefore predicted that (a) distractor words would now interfere with responding to arrows, and (b) distractor arrows would continue to interfere with responding to words but would now do so more strongly than in Experiment 1. We further expected the boost in interference to be roughly equivalent for the two types of stimuli, given the same increased amount of target uncertainty and consequently more complex task set. We consider the rationale for these predictions at greater length in the introduction to Experiment 2.

## Experiment 1

### Method

#### Participants

**Figure 1.**
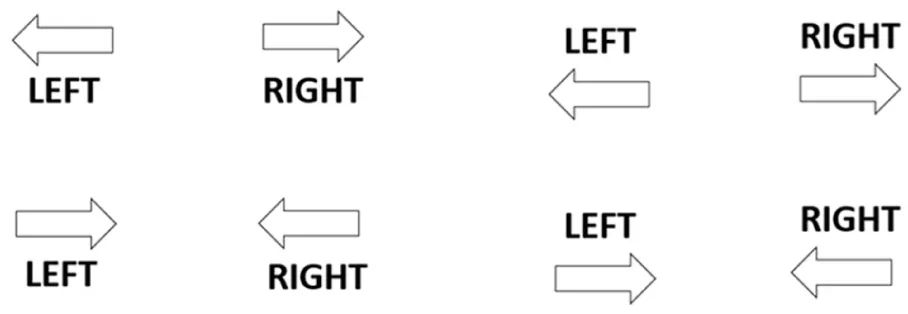
The stimuli used in Experiments 1 and 2.

A total of 118 participants were recruited through the Department of Psychology’s SONA pool. All were University of Waterloo undergraduate students who reported normal or corrected-to-normal vision and who participated in exchange for partial course credit. Of these 118 participants, 60 saw displays in which the stimuli consisted of an arrow presented above a directional word (the four stimuli on the left in Figure 1); 58 saw displays in which an arrow was presented below a directional word (the four stimuli on the right in Figure 1). We refer to this between-subjects variable as Stimulus Location. The two experiments were approved by the Office of Research Ethics (REB #45020) at the University of Waterloo.

#### Stimuli

The eight stimuli, consisting of type of stimulus (word vs. arrow), relation of the two elements (congruent vs. incongruent), and location of the two elements (word on top vs. arrow on top), are shown in Figure 1. The arrow was the same width as the word *RIGHT* and the same height as the word stimuli (2.9 cm × 1.45 cm). All arrows were presented in white with a black outline 0.75-point wide. In both experiments, words were presented in black, Calibri bold, 32-point font. Stimuli were presented in the center of a 5 cm × 5 cm box with black edges presented on a white background.

#### Programming

The experiment was created using PsychoPy (Peirce et al., 2019) and was hosted on their online platform Pavlovia (https://pavlovia.org/). In Pavlovia, when a participant follows the link to the experiment, displays are automatically presented in full screen, preventing the use of other functions of the browser (e.g., clicking on different tabs, minimizing or closing the browser) unless the participant were to press the F11 key (that would allow the experimenter to exit the program), which never happened.

#### Procedure

Participants followed a link to the experiment on Pavlovia where they read the instructions presented on their screen. Depending on the block, they were instructed to respond either to the direction pointed to by the arrow or to the direction indicated by the word, in both cases by using the keys “Q” for left and “P” for right using their two index fingers. Task order (respond to the arrow vs. respond to the word) was counterbalanced across blocks.

Each block began with 12 practice trials that were faithful to the experimental trials (3 each of the 4 possible pairings) except that, unlike during the experimental trials, feedback was provided after each trial. The feedback consisted of the word CORRECT or INCORRECT displayed in black uppercase, 18-point, Arial font for 1 s. Practice was followed by 80 experimental trials, 40 congruent and 40 incongruent in random order, for each block.

Each trial began with a 500-ms blank screen, followed by the edged black box displayed for 300 ms. The display then appeared in the center of the box and remained there until the participant made a response. After the participant made a response, an X appeared in the center of the screen as a signal to the participant to press the spacebar with their thumb to initiate the next trial. Participants were instructed to keep their two index fingers on the two response keys throughout.

### Results and Discussion

A limitation of experiments conducted on online platforms is that some participants do not pay close attention, especially to the typical instruction to “respond as quickly as possible but try not to make too many errors.” Indeed, our experience is that between 10% and 20% of participants commit numerous errors in one or more conditions. Consequently, we tested more participants than we typically would for experiments conducted in the laboratory, expecting to discard roughly that proportion. Guided by previous work (Besner & Young, 2022), we discarded the data of participants who made 20% or more errors in any one condition. This led to the data of 10 participants (8.5%) being discarded.^4^

Trials on which an error was committed were discarded, which led to a total of 2.2% of trials being removed. Next, for each participant, correct response times beyond 2.5 standard deviations above or below the mean in that condition were discarded as recommended by Van Selst and Jolicoeur (1994). This led to removal of 2.7% of trials. Finally, a mean for each condition for each participant was calculated, and the data of any participant whose mean fell more than 3 standard deviations from the grand mean of that condition were discarded. This led to the data of four participants (3.4%) being removed. Thus, after removal of the data of 14 participants in total, 104 participants remained for analysis. Removal of the data of 11.9% of participants is quite typical for online experiments. We note that the sample size in our experiment was almost three times that of Baldo et al. (1998).

Mean percent error and correct mean response time are shown in Table 1. For both dependent measures, initial 2 × 2 × 2 mixed analyses of variance (ANOVA), with the factors of Task (word vs. arrow), Congruency (congruent vs. incongruent), and Stimulus Location (word on top vs. arrow on top; between subjects), revealed no main effect of Stimulus Location nor did Stimulus Location enter into any interactions (all *Fs* < 1). We therefore collapsed over Stimulus Location and report simpler 2 × 2—Task × Congruency—repeated measures ANOVAs.

**Table 1.** Experiment 1: Mean Response Time (RT; ms) and Mean Percent Error as a Function of Task and Congruency.

Congruency	Respond to arrows		Respond to words	
	RT	% Error	RT	% Error
Incongruent	418 (6)	1.2 (0.2)	527 (8)	4.9 (0.4)
Congruent	417 (5)	1.2 (0.2)	482 (7)	1.4 (0.2)
Congruency effect (difference)	1	0.0	45	3.5

*Note.* Numbers in parentheses represent standard error. RT: response time.

In anticipation of a null interference effect of words on responding to arrows, we also report Bayes factors for the congruency effect *t*-tests. For those unfamiliar with Bayesian statistics, a *BF*_01_ denotes the level of support for the null hypothesis being true; in contrast, a BF_10_ denotes the level of support for the alternative hypothesis being true. The Bayes factors reported here were calculated using JASP vers 0.18.3 (JASP Team, 2024). Lee and Wagenmakers (2014) label a Bayes factor below 3 as anecdotal evidence, from 3 to 10 as moderate evidence, from 10 to 30 as strong evidence, from 30 to 100 as very strong evidence, and beyond 100 as extreme evidence.

#### Percent Error

There were significant main effects of both Task, *F*(1,103) = 61.05, *MSE* = 0.0007, *p* < .001, ŋ_*p*_^2^ = .372, and Congruency, *F*(1,103) = 51.48, *MSE* = 0.0006, *p* < .001, ŋ_*p*_^2^ = .333. The central finding of interest was the significant Congruency by Task Interaction, *F*(1,103) = 65.35, *MSE* = 0.0005, *p* < .001, ŋ_*p*_^2^ = .388. The 3.5% congruency effect for responding to words was reliable, *t*(103) = 8.54, *p* < .001, *d* = 0.84, *BF*_10_ = 5.83 × 10^10^; there was no congruency effect for responding to arrows (a 0% difference) with moderate evidence for the null, *BF*_01_ = 9.15.

#### Correct Response Time

There were significant main effects of both Task, *F*(1,103) = 214.78, *MSE* = 3,653.72, *p* < .001, ŋ_*p*_^2^ = .676, and Congruency, *F*(1,103) = 110.79, *MSE* = 512.69, *p* < .001, ŋ_*p*_^2^ = .518. Again, the central finding of interest was the significant Congruency by Task Interaction, *F*(1,103) = 113.88, *MSE* = 448.17, *p* < .001, ŋ_*p*_^2^ = .532. Although distractor arrow direction substantially interfered with judging the direction represented by the word (45 ms), *t*(103) = 11.60, *p* < .001, *d* = 1.14, *BF*_10_ = 2.39 × 10^17^, there was virtually no interference from distractor words when judging arrow direction (1 ms), with moderate evidence for the null, *BF*_01_ = 7.97.

The results are clear in showing that distractor arrows strongly interfered with directional judgments to words, whereas distractor words did not interfere with directional judgments to arrows. This pattern replicates that reported by Baldo et al. (1998) for manual responses. Indeed, the response times and error rates were very similar across the two studies. These findings are, therefore, entirely consistent with the S–R compatibility account of Baldo et al. They also fit with the idea that task set is very simple when trials are blocked such that task set is straightforward across an entire block of trials. The complete absence of interference from words when responding to arrows remains surprising, however, given our extensive experience with the processing of words.

## Experiment 2

In essence, blocking trials by target stimulus in Experiment 1 resulted in a simple task set where a target was always a target and a distractor was always a distractor. This allowed participants to minimize interference from the word in responding to the arrow because the word was always a distractor and could quite easily be ignored. Experiment 2 sought to demonstrate how a change in context resulting in a more complex task set would alter this pattern with the consequence that distractor words now would interfere when arrows were the target because with task set potentially changing from trial to trial, the words could not now be easily ignored.

Experiment 2 again presented participants with an arrow and a word on each trial. Critically, however, task set was made more complex than in Experiment 1 in that the target now was defined by location. Now, on a random half of the trials, the word was located above the arrow; on the other half of the trials, the word appeared below the arrow. Half of the participants were instructed to respond to the upper stimulus while ignoring the lower one; the other half of the participants were instructed to respond to the lower stimulus while ignoring the upper one. This creates a more complex task set in that a target now is sometimes a word and sometimes an arrow and a distractor is also sometimes a word and sometimes an arrow.

Regardless of the context, the optimal strategy is of course to ignore the distractor as much as possible. From a task set perspective, this was relatively easy in our Experiment 1 and in Experiment 1 of Baldo et al. (1998) where one stimulus type was always the one to be responded to—always an arrow or always a word, depending on the block—and the other stimulus type was always to be ignored. In Experiment 2, however, ignoring the distractor will be harder because now location determines which stimulus to respond to—always the top stimulus or always the bottom stimulus. Thus, from trial to trial, the target could be either a word or an arrow. This constitutes inconsistency over trials requiring greater control because of a more complex task set.

This analysis leads to two straightforward predictions. First, interference should now be observed from words when judging the direction of arrows because focusing exclusively on arrows cannot be done consistently from trial to trial. Second, this same inconsistency across trials should result in increased interference observed from arrows when judging the direction indicated by a word. With the more complex task set of Experiment 2, interference should increase for both stimulus types, becoming reliable for words interfering with arrows and growing (relative to Experiment 1) for arrows interfering with words.

### Method

#### Participants

Sixty-five undergraduates from the University of Waterloo participated in exchange for partial course credit through the SONA participant pool. All reported having normal or corrected to normal vision. Participants in one group (*N* = 32) were instructed to respond to the direction of an arrow or word that appeared above the distractor; participants in the other group (*N* = 33) were instructed to respond to the direction of an arrow or word that appeared below the distractor. We refer to this between-subjects variable as Response Position.

#### Stimuli and Apparatus

The stimuli were the same as in Experiment 1 except that now an arrow appeared above the word on 50% of the trials and below the word on 50% of the trials.

#### Procedure

Participants were tested in a room with multiple testing stations. They first read the instructions presented on the screen. Then, the experiment started with 24 practice trials in which participants received feedback after each practice trial, as in Experiment 1. These practice trials were then followed by the 136 experimental trials, half congruent and half incongruent. In this experiment, the display on any given trial could involve the same target element as the preceding trial or it could involve the other target element. Thus, the target task varied randomly across trials. Task repetition would be expected to speed responding, so we added a variable to the analysis to capture this: Target Transition denotes whether the target on a given trial *N* + *1* was the same as that on trial *N*—a “stay” trial—or was different from that on trial *N*—a “shift” trial. As it turned out, the only difference between these two types of trials was that responses on stay trials were considerably faster than those on shift trials—a main effect without interactions.

### Results and Discussion

The data were first subjected to the same screening procedure as in Experiment 1. Trials on which an error was committed were discarded for a total of 2.8% of all trials. This led to the data of 14 participants (21.5%; 7 in each group) being removed due to having 20% or greater errors in any one condition. Next, correct response times beyond 2.5 standard deviations above or below, the mean in any condition were discarded; this led to a further 3.0% of all trials being removed. The data from 51 participants (25 in the respond-to-top group and 26 in the respond-to-bottom group) remained for analysis.

Correct mean response time and mean percent error are shown in Table 2. For both dependent measures, initial 2 × 2 × 2 × 2 mixed ANOVAs, with the factors of Task (word vs. arrow), Congruency (congruent vs. incongruent), Target Transition (stay vs. shift), and Response Position (top vs. bottom; between-subjects), revealed no significant effects involving Response Position. For percent error, neither the main effect of Response Position, *F*(1,49) = 1.67, *MSE* = 0.003, *p* = .202, ŋ_*p*_^2^ = .033, nor any interactions involving Response Position (all *Fs* < 1) approached significance. For response time, the main effect of Response Position was not significant, *F* < 1, and only the non-significant interaction of Task × Response Position, *F*(1,49) = 3.35, MSE = 10,526.43, *p* = .074, ŋ_*p*_^2^ = .064, exceeded *F* = 1. We therefore removed Response Position and report simpler 2 × 2 × 2—Task × Congruency × Target Transition—ANOVAs. Again, we report Bayes factors for all congruency effect *t*-tests.

**Table 2.** Experiment 2: Mean Response Time (RT; ms) and Mean Percent Error as a Function of Task, Congruency, and Stay/Shift.

Congruency	Respond to arrows				Respond to words			
	Stay		Shift		Stay		Shift	
	RT	% Error	RT	% Error	RT	% Error	RT	% Error
Incongruent	653 (28)	2.2 (0.6)	749 (26)	3.7 (1.1)	717 (29)	3.1 (0.7)	802 (31)	10.6 (1.3)
Congruent	594 (26)	0.4 (0.3)	655 (25)	0.6 (0.3)	608 (23)	0.8 (0.1)	658 (28)	0.6 (0.3)
Congruency effect (difference)	59	1.8	94	3.1	109	2.3	144	10.0

*Note.* Numbers in parentheses represent standard error. RT: response time.

#### Percent Error

All three main effects were significant: Task, *F*(1,50) = 13.05, *MSE* = 0.003, *p* < .001, ŋ_*p*_^2^ = .207, Congruency, *F*(1,50) = 83.20, *MSE* = 0.002, *p* < .001, ŋ_*p*_^2^ = .625, and Target Transition, *F*(1,50) = 33.57, *MSE* = 0.002, *p* < .001, ŋ_*p*_^2^ = .402. All interactions were also significant: Task × Congruency, *F*(1,50) = 21.58, *MSE* = 0.002, *p* < .001, ŋ_*p*_^2^ = .301, Task × Target Transition, *F*(1,50) = 13.33, *MSE* = 0.002, *p* < .001, ŋ_*p*_^2^ = .211, Congruency × Target Transition, *F*(1,50) = 26.59, *MSE* = 0.002, *p* < .001, ŋ_*p*_^2^ = .347, and Task × Congruency × Target Transition, *F*(1,50) = 11.71, *MSE* = 0.002, *p* < .001, ŋ_*p*_^2^ = .190. As in Experiment 1, the central finding of interest is the significant Task by Congruency interaction. Here, the 6.4% congruency effect for responding to words was reliable, *t*(50) = 8.32, *p* < .001, *d* = 1.17, *BF*_10_ = 1.82 × 10^8^, as was the 2.5% congruency effect for responding to arrows, *t*(50) = 4.73, *p* < .001, *d* = 0.66, *BF*_10_ = 1,053.17. The congruency effect was greater for responding to words than to arrows, *t*(50) = 4.36, *p* < .001, *d* = 0.61, *BF*_10_ = 339.89.

#### Correct Response Time

All three main effects were significant: Task, *F*(1,50) = 10.48, MSE = 11,020.08, *p* = .002, ŋ_*p*_^2^ = .173, Congruency, *F*(1,50) = 8,126.45, *MSE* = 8,308.77, *p* < .001, ŋ_*p*_^2^ = .717, and Target Transition, *F*(1,50) = 82.06, *MSE* = 6,678.92, *p* < .001, ŋ_*p*_^2^ = .621. Of the interactions, only two were significant: Task × Congruency, *F*(1,50) = 20.86, *MSE* = 3,144.77, *p* < .001, ŋ_*p*_^2^ = .294, and Congruency × Task Transition, *F*(1,50) = 11.28, MSE = 2,673.96, *p* = .002, ŋ_*p*_^2^ = .184. Task × Task Transition and the 3-way interaction both were nonsignificant (*F*s < 1). Again, the central finding of interest is the significant Task by Congruency interaction. The 126 ms congruency effect for responding to words was reliable, *t*(50) = 10.32, *p* < .001, *d* = 1.45, *BF*_10_ = 1.359 × 10^11^, as was the 76 ms congruency effect for responding to arrows, *t*(50) = 8.24, *p* < .001, *d* = 1.50, *BF*_10_ = 1.379 × 10^8^. Responding to words showed greater interference than did responding to arrows, *t*(50) = 3.52, *p* < .001, *d* = 0.49, *BF*_10_ = 29.96.

### Cross-Experiment Comparisons for Percent Error

#### Stay Trials

A Congruency × Experiment ANOVA for word distractors when responding to arrows confirmed that, unlike in Experiment 1, there was a congruency effect in Experiment 2, *F*(1,153) = 13.44, *MSE* = 0.0004, *p* < .001, ŋ_*p*_^2^ = .085, *BF*_10_ = 66.32. The Congruency × Experiment ANOVA for arrow distractors when responding to words was not significant, *F*(1,153) = 0.45, *MSE* = 0.001, *p* = .501, ŋ_*p*_^2^ = .003, *BF*_01_ = 4.10.

#### Shift Trials

A Congruency × Experiment ANOVA for word distractors when responding to arrows confirmed that, unlike in Experiment 1, there was a congruency effect in Experiment 2, *F*(1,153) = 21.35, *MSE* = 0.0007, *p* < .001, ŋ_*p*_^2^ = .122, *BF*_10_ = 2,881.95. The Congruency × Experiment ANOVA for arrow distractors when responding to words confirmed that the congruency effect was larger in Experiment 2 than in Experiment 1, *F*(1,153) = 37.97, *MSE* = 0.002, *p* < .001, ŋ_*p*_^2^ = .199, *BF*_10_ = 5.176 × 10^6^.

### Cross-Experiment Comparisons for Correct Response Time

#### Stay Trials

A Congruency × Experiment ANOVA for word distractors when responding to arrows confirmed that, unlike in Experiment 1, there was a congruency effect in Experiment 2, *F*(1,153) = 55.68, MSE = 1,036, *p* < .001, ŋ_*p*_^2^ = .267, *BF*_10_ = 8.049 × 10^8^. A parallel Congruency × Experiment ANOVA for arrow distractors when responding to words confirmed that the congruency effect was larger in Experiment 2 than in Experiment 1, *F*(1,153) = 31.28, MSE = 2,225, *p* < .001, ŋ_*p*_^2^ = .170, *BF*_10_ = 71,990.55.

#### Shift Trials

A Congruency × Experiment ANOVA for word distractors when responding to arrows confirmed that, unlike in Experiment 1, there was a congruency effect in Experiment 2, *F*(1,153) = 121.36, MSE = 1,202, *p* < .001, ŋ_*p*_^2^ = .442, *BF*_10_ = 2.012 × 10^17^. A parallel Congruency × Experiment ANOVA for arrow distractors when responding to words confirmed that the congruency effect was larger in Experiment 2 than in Experiment 1, *F*(1,153) = 73.72, MSE = 2,243, *p* < .001, ŋ_*p*_^2^ = .352, *BF*_10_ = 2.205 × 10^11^.

In summary, we correctly predicted that Experiment 2 would yield a congruency effect for word distractors when responding to arrows that was not evident in Experiment 1, and that the effect for arrow distractors when responding to words would be larger in Experiment 2 than in Experiment 1. The larger congruency effect here could also be a reflection of the response congruity effect often observed in task switching (see, e.g., Yamaguchi & Proctor, 2011) or of more diffuse attentional selection under uncertainty leading to more extensive processing of the distractor dimension, although we will argue that this pattern supports our task set explanation. Overall, the observed data pattern is consistent with the prediction that the more complex task set in Experiment 2 than in Experiment 1 would introduce interference caused by incongruent words when judging the direction of arrows and would enhance the interference caused by incongruent arrows when judging the direction indicated by words.

## General Discussion

The first question that these experiments sought to answer was whether the identification of the words LEFT and RIGHT would be protected from potential interference caused by incongruent arrows in the display because, unlike arrow processing. word processing is prioritized due to its extensive practice. Would the findings of Baldo et al. (1998) that contradicted this expectation be replicated? The results from Experiments 1 and 2 (and from the experiment described in footnote 1) confirm those of Baldo et al., showing clearly that such protection is not afforded. Words, like schematic eyes in Besner et al. (2021), are not generally prioritized: Instead, their processing depends on the context in which they are embedded—the task set.

The second question concerned the extent to which visual word recognition would, when incongruent, nonetheless be capable of interfering with another process, here that of determining the direction of an arrow. The results of Baldo et al. (1998) and of Experiment 1 clearly showed that the words LEFT and RIGHT failed to affect the imperative arrow task. The small, nonsignificant effect of words on arrows in their Experiment 1 vanished altogether in our higher-powered Experiment 1. As predicted, however, distractor words did indeed interfere with judging arrow direction in Experiment 2 when identification of the target was made more difficult by being inconsistent from trial to trial, requiring that a more complex task set be held in working memory.

The third question concerned the sufficiency of the Baldo et al. (1998) S–R compatibility account of the finding that (at least for manual responding) arrows interfere with words but words do not interfere with arrows. On their account, the mapping of arrows to manual responses is S–R compatible; hence, arrows should produce interference with processing the word’s meaning. The mapping between words and manual responses is, however, much less S–R compatible and hence words, when incongruent, should not produce interference. This pattern is precisely what they and we observed. The S–R compatibility account was sufficient when considering only their results and our Experiment 1 replication of those results.

One of the most remarkable things about S–R compatibility in the case of arrows is just how prevalent their influence is, given that arrows are irrelevant across an entire block of trials both in Baldo et al. (1998) and in our Experiment 1. In these circumstances, the large amount of interference caused by an arrow in responding to a word likely reflects direct activation of response codes that have particularly high “set-level” compatibility with their responses (Haazebroek et al., 2017; Kornblum et al., 1990).

The difficulty with the S–R explanation arises in Experiment 2, where arrows and words both caused interference with each other despite a manual response still being required for both tasks. Because the S–R mappings for the two tasks in Experiment 2 were the same as in Experiment 1, the Baldo et al. (1998) S–R compatibility account fails: It provides no explanation for the findings of Experiment 2—incongruent words now interfering with arrows coupled with arrows now interfering more with words than in Experiment 1.

Unlike in Experiment 1, where a single task set applied throughout a block of trials, in Experiment 2 both task sets had to be maintained to make rapid switching possible based on the display on a given trial. Maintaining both word and arrow task sets provides a straightforward account of one of the central results from Experiment 2. Interference of words on arrows emerged because the task set for words was now maintained at an above-threshold activation level. By contrast, in Experiment 1, in the block of trials where the arrows task was always relevant (i.e., performed) and the words were always irrelevant, the task set for words was inactive, hence the null effect of distractor words on arrows.

It also follows that what should be seen in Experiment 2 is an increase in the amount of interference from an incongruent arrow on a judgment about the word as compared to Experiment 1. The task set for arrows is now more active than in Experiment 1 where it was a distractor throughout the block of trials that required responding to words. This cross-experiment effect is evident; in that the amount of interference from arrows is larger in Experiment 2 (126 ms averaged across stay and shift trials) than in Experiment 1 (45 ms). Indeed, Table 3 shows that the more complex task set in Experiment 2 resulted in a roughly equivalent increase in the congruency effect (incongruent—congruent) both for arrows on words and for words on arrows.^5^

**Table 3.** Mean Congruency Effect (Incongruent–Congruent) in ms as a Function of Task and Experiment.

Experiment	Respond to arrows	Respond to words
Experiment 1	1	45
Experiment 2 (stay)	59	109
Experiment 2 (shift)	94	144

One might argue that what explains the change in moving from Experiment 1 to Experiment 2 is the introduction of task switching—not the increase in task set complexity—in Experiment 2. It is certainly true that differentiating a change in task set from a change in task switching is difficult. One feature in particular of our data pattern is, however, more consistent with our task set account than with a task-switching account. By definition, stay trials do not involve a shift yet in Experiment 2 the stay trials showed largely the same pattern as the shift trials. If these results were all about task switching, then the pattern for stay trials in Experiment 2 should have been the same as that for all trials in Experiment 1—where all trials were stay trials—but it was not.

Overall, the pattern of results observed here shows that the Baldo et al. (1998) S–R compatibility account, although an important piece of the story, is insufficient precisely because changing the task set without changing the response mode or the stimuli strongly altered the data pattern. The more elaborated account offered here, in which the complexity of the task set is critical, provides a sufficient account of all of the data. The results observed here also remind us of a more general and important point: Theoretical accounts of processing, even in the context of such simple judgments as those considered here, must recognize that task set is an important piece of the puzzle.

## Notes

1. That said, major changes in the relative size of the eyes and arrows might well be expected to reverse the pattern of interference. Besner et al. (2021) discussed this and pointed to several lines of evidence suggesting that, at least with respect to their eye and arrow stimuli, this was not an issue. This same constraint holds in the experiments reported here using arrows and words. Notably, we also conducted an experiment in which the arrow was made very much smaller than the word, and we observed the same pattern and magnitude of interference: There was no interference in the processing of arrows due to incongruent words, but there was strong interference in the processing of words due to incongruent arrows.

2. In fact, depending on the physical layout of the display, incongruent words can interfere with arrows (Luo & Proctor, 2013; Shor, 1970, among others). For example, this can occur when the incongruent word appears inside an outline of an arrow, where spatial integration makes the word much harder to ignore (Shor, 1970). Whether the targets or their locations are certain will also be influential (Luo & Proctor, 2013). These results likely speak to the importance of salience, integration, and/or attentional capture.

3. One way to think about this pattern is to see the processing of arrows and words as different architecturally: Arrows denote direction conceptually, whereas words are more tied to their sounds and from there to the corresponding concepts (see the arguments of Roelofs, 2003).

4. To facilitate the removal of participants who did not complete the study, we opted out of the “save incomplete data” option on Pavlovia. In addition, to receive credit, participants had to complete the entire study (or in the case of a mid-study withdrawal, they would contact us as per TCPS 2-CORE Guidelines and the ethical guidelines employed at the University of Waterloo; no such incident occurred).

5. Table 3 also shows a virtually constant 50-ms benefit for stay versus shift trials in Experiment 2.
